# Chitosan-Based Schiff Base Compounds: Synthesis, Chemical Characterization and Antibacterial Properties

**DOI:** 10.1007/s10895-025-04233-x

**Published:** 2025-04-11

**Authors:** Nuran Çelikçi, Cengiz Ayhan Zıba, Mehmet Tümer

**Affiliations:** 1 Department of Material Science and Engineering, Institute of Science and Technology, Kahramanmaras Sutcu Imam University, Kahramanmaras, Turkey; 2 Department of Chemical Technologies, Afsin Vocational School, Kahramanmaras Sutcu Imam University, Kahramanmaras, Turkey; 3 Chemistry Department, Faculty of Science, Kahramanmaras Sutcu Imam University, Kahramanmaras, Turkey

**Keywords:** Chitosan Schiff-base, NMR, Antibacterial properties

## Abstract

The focus of the study was the synthesis, characterization, and investigation of the antibacterial properties of chitosan Schiff bases (CHSB) and crosslinked chitosan Schiff bases (CCHSB) resulting from the reaction of chitosan with 2,3-dihydroxybenzaldehyde and epichlorohydrin. The physicochemical properties of the synthesized compounds were analyzed using 1H NMR, FTIR, XRD, SEM, and TGA techniques. The chemical shifts of chitosan appeared between 1.78 and 4.71 ppm. Aromatic ring protons from the Schiff base appeared between 6.68 and 7.79 ppm. Epichlorohydrin methylene protons for crosslinking were observed as triplets in the range of 1.24–1.04 ppm in the 1 H-NMR spectrum of cross-linked Schiff bases. The antibacterial activity of the chitosan and Schiff base derivatives on gram-negative bacteria (*Escherichia coli and Salmonella typhimurium*) and gram-positive bacteria (*Staphylococcus aureus and Bacillus cereus*) was compared with the drugs amikacin and gentamicin. Results showed that the chitosan derivatives exhibited antibacterial properties, with inhibition zones ranging from 8 to 30 mm. Chitosan derivatives demonstrated antibacterial activity comparable to standard drugs like amikacin and gentamicin, suggesting their potential as effective alternatives or adjuncts in treatments. These findings indicate that chitosan and its derivatives have the potential to be utilized as effective antibacterial agents or alternatives in a variety of medical domains. Additionally, chitosan derivatives may serve as natural preservatives in the food industry by inhibiting microbial growth, thereby extending the shelf life of perishable products and ensuring their quality and safety.

## Introduction

Chitosan, a biopolymer derived from the deacetylation of chitin (β-(1 → 4)-2-amino-2-deoxy-D-glucopyranose), is recognized for its high chelating capacity due to the presence of numerous primary amino groups distributed regularly along its molecular chains [1–3]. Chitosan, as one of the most abundant natural polysaccharides after cellulose, possesses unique properties such as biocompatibility, biodegradability, and non-toxicity, which make it suitable for a wide array of applications [4]. While chitosan is insoluble in water, it can dissolve in aqueous solutions of organic acids such as acetic acid, formic acid, citric acid, and diluted hydrochloric acid, forming viscous solutions [5]. Chitosan has become a subject of significant interest due to its cationic structure, biological activities including antioxidant, antitumor, and antimicrobial properties, biocompatibility, biodegradability, and non-toxic nature [6–8]. The hydroxyl and amino functional groups in the structure of chitosan allow chemical modification to improve its physicochemical properties. The amino groups can readily react with aldehydes and ketones to form Schiff bases (CH = N), a class of organic compounds characterized by the presence of an imine (azomethine) group (-C = N) [9]. The reaction of chitosan with aldehydes or ketones results in the formation of aldimines or ketoimines, respectively, through condensation, where water is eliminated [10]. Schiff base derivatives of chitosan are typically synthesized by condensing the amino groups of chitosan with the carbonyl groups of aldehydes or ketones, resulting in a condensation product. In a neutral medium, chitosan reacts readily with aromatic or aliphatic aldehydes and ketones to form Schiff bases. The synthesis of CHSBs can be facilitated using various solvents, including acetic acid, ethanol, methanol, or a combination of these solvents [11]. Additionally, solvents such as dimethylformamide (DMF) [12] water [13], and ionic liquids [14] have also been employed in the synthesis of CHSBs. Schiff base derivatives of chitosan are widely utilized in a variety of applications, including as pigments and dyes, catalysts, polymer stabilizers, luminescent chemosensors, and intermediates in organic synthesis [15–17]. Schiff base derivatives of chitosan are one of the most significant modifications and the newly formed imine groups improve the pharmacological actions of chitosan such as antitumor, antibacterial, and antioxidant [11]. One of the chemical modifications of chitosan is cross-linking, which is employed to improve several of its properties, including acid stability, mechanical strength, pore size, hydrophilicity, and biocompatibility [18–22]. Cross-linking agents, such as dialdehydes (e.g., glutaraldehyde), epichlorohydrin (ECH), and ethylene glycol diglycidyl ether (EDGE), are commonly used in combination with chitosan to enhance these properties [23–26].

The antimicrobial evaluation of chitosan and its derivatives, particularly CHSB, has revealed significant potential for the development of effective antimicrobial agents. Several studies have demonstrated that chitosan derivatives, especially those modified into Schiff bases, exhibit enhanced antibacterial and antifungal activities against a variety of pathogenic microorganisms [27–30]. Schiff bases derived from functionalized chitosan, such as those incorporating heteroaryl pyrazole derivatives, have shown considerable antimicrobial efficacy against a range of pathogens, including *Streptococcus mutans*, *Staphylococcus aureus*, *Escherichia coli*, *Klebsiella pneumoniae*, *Candida albicans*, and *Aspergillus fumigatus* [31–32].

Building on these findings of chitosan and Schiff base derivatives, the present article focuses on the synthesis and antimicrobial properties of CHSB and CCHSB, synthesized using chitosan and aromatic aldehydes, specifically 2,3-dihydroxybenzaldehyde. Both CHSB and CCHSB were synthesized and their structures were characterized using variety of spectroscopic techniques, including Fourier-transform infrared spectroscopy (FTIR), nuclear magnetic resonance (NMR) spectroscopy, and X-ray diffraction (XRD). In addition, the surface morphologies and thermal properties were examined using scanning electron microscope (SEM), and thermogravimetric analysis (TGA), respectively. These comprehensive characterizations provide valuable information insights into the structural, morphological, and thermal properties of the synthesized chitosan derivatives and their potential as antimicrobial agents.

## Experimental Study

### Materials

Powdered chitosan (degree of deacetylation 75–85%), sodium hydroxide (NaOH, 99%), hydrochloric acid (HCl, 37%), glacial acetic acid (CH₃COOH), isopropyl alcohol (IPA), ethanol, methanol, and acetone were procured from Sigma-Aldrich. All chemicals utilized in this study were of analytical grade.

### Synthesis of the CHSB and CCHSB

**Scheme 1:**
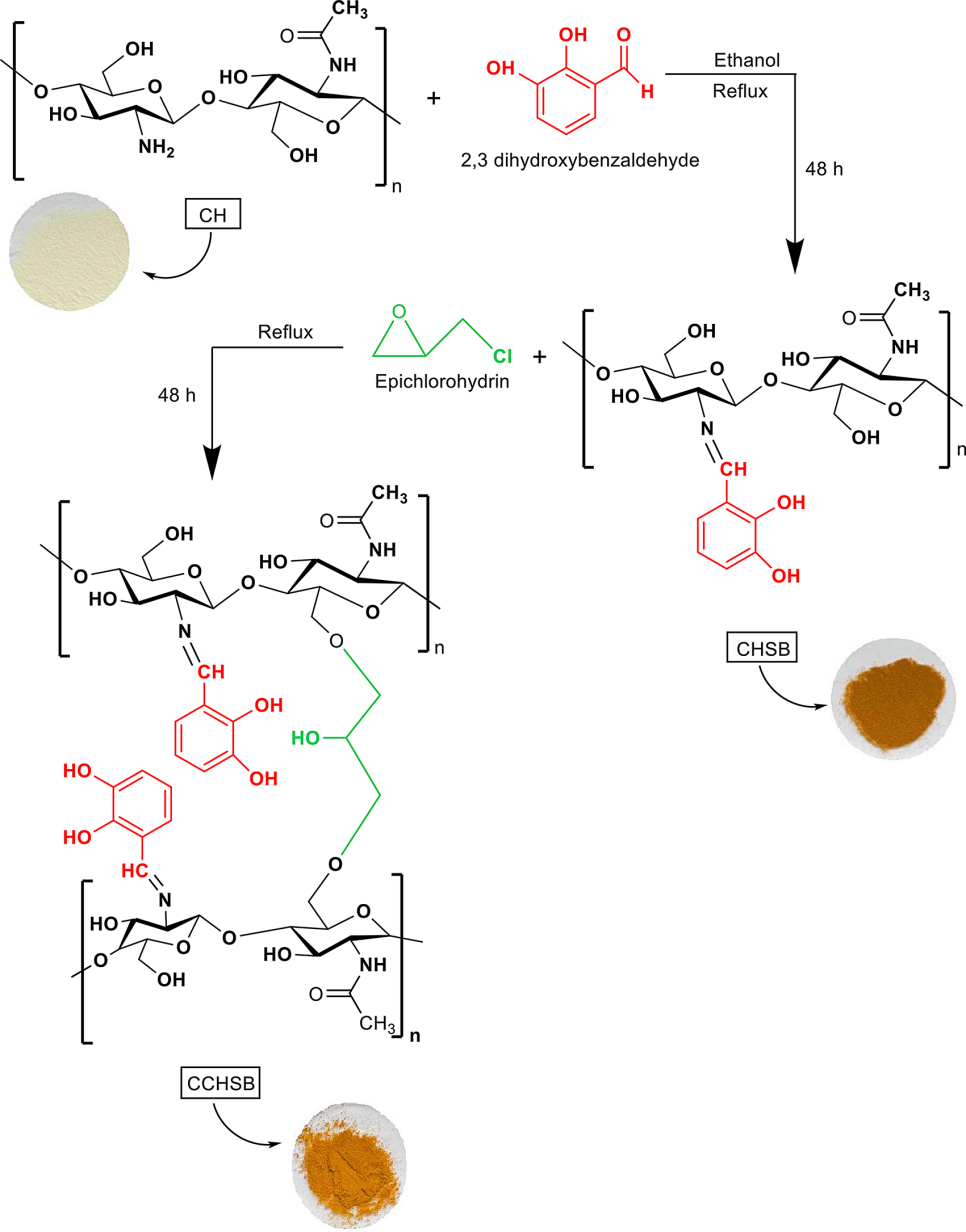
Synthesis reaction of chitosan and Schiff base derivatives

The CHSB was synthesized according to a reference method with some modifications [33]. To prepare the Schiff base, 0.54 g (0.003 mol) of chitosan was dissolved in %1 acetic acid and 25 mL of ethanol and continuously stirred for 30 min. Subsequently, 0.82 g (0.006 mol) of 2,3-dihydroxybenzaldehyde was added to the solution, and the mixture was stirred at 70 °C for 4 days. After the reaction, the solution was allowed to cool to room temperature, filtered, washed with ethanol and acetone, and the product was isolated as a yellow powder after drying at room temperature. Following the preparation of CHSB, crosslinking was performed by adding 0.32 mL (0.003 mol) of epichlorohydrin to the reaction mixture to yield crosslinked chitosan Schiff base. After completion of the reaction, the solution was filtered at room temperature, washed several times with diethyl ether to remove residual crosslinker agent, and dried at room temperature. A schematic representation of the synthesis of CHSB and CCHSB is provided in Scheme 1.

### Characterization Approaches

The morphology of the synthesized samples was observed using a SEM (JEAL / NEOSCOPE JCM-5000) at an accelerating voltage of 20 kV. The FT-IR spectra of the recorded in the range of 4000 to 400 cm⁻¹ using a Perkin Elmer Spectrum 400 Infrared Spectrometer equipped with an ATR apparatus. The ¹H NMR spectra were obtained at 30 °C on a Bruker-200 MHz Varian spectrometer, employing a 90° pulse and 16 scans. The samples were dissolved in CD_3_COOD/D_2_O solution at a concentration of 25–30 mg/600 µL with chemical shifts reported in ppm. X-ray diffraction (XRD) patterns of the samples were recorded using a Philips X’Pert PRO diffractometer, operating with CuKα radiation, a voltage of 40 kV, and a current of 30 mA. All samples were scanned in the range of 10° to 90° 2θ at a scanning speed of 5^o^ 2θ /min with a step size of 0.02°. The thermal properties of the samples were measured by using TG-DTA (SEIKO II, Seiko, Japan). The sample (15 ± 5 mg) was placed in a ceramic dish and heated from 30 °C to 600 °C at a heating rate of 20 °C/min under a nitrogen flow (20 mL/min).

### In Vitro antibacterial Assays

The antibacterial activities were evaluated using the Kirby-Bauer disc diffusion method and the minimum inhibitory concentration (MIC) method. These methods were employed to investigate the antibacterial effects of chitosan and its derivatives against two gram-negative bacteria (*Escherichia coli*, ATCC: 8739 and *Salmonella Typhimurium*, ATCC: 14028), two gram-positive bacteria (*Staphylococcus aureus*, ATCC: 6538 and *Bacillus cereus*, ATCC: 7064), and *Candida albicans* (ATCC: 90028). The bacterial pathogens were subcultured in Mueller-Hinton Broth (MHB; 1% peptone, 0.5% yeast extract, 1% NaCl, pH 7 ± 0.2) and inoculated onto MHB and Mueller-Hinton Agar (MHA) using sterile cotton swabs, followed by incubation at 37 ± 1 °C for 18 h. Sterile paper discs (Whatman No. 2668, 6 mm diameter) were impregnated with 10 µL of crude extract solution, placed on the MHB and MHA media, and incubated at 37 °C for 18–24 h. All bacterial cultures were adjusted to 0.5 McFarland standards. Discs containing amikacin (AK: 30 µg) and gentamicin (CN: 10 µg) in dimethyl sulfoxide (DMSO) were used as controls. The diameter of the inhibition zone (in mm) surrounding each disc was measured. All experiments were performed in triplicate to ensure accuracy and reproducibility.

#### Determination of Minimum Inhibitory Concentration (MIC) and Inhibition Zone Diameter (IZD)

The MIC method was employed as a quantitative approach to assess the antibacterial activity of the tested compounds against the selected organisms [34]. The effect of varying concentrations of CH, CHSB, and CCHSB (12.5 mg/mL, 1.25 mg/mL, and 0.125 mg/mL) on the growth of *E. coli*, *Salmonella Typhimurium*, *Staphylococcus aureus*, and *Bacillus cereus* was investigated using this method.

## Result and Discussion

### SEM Analysis

**Fig. 1:**
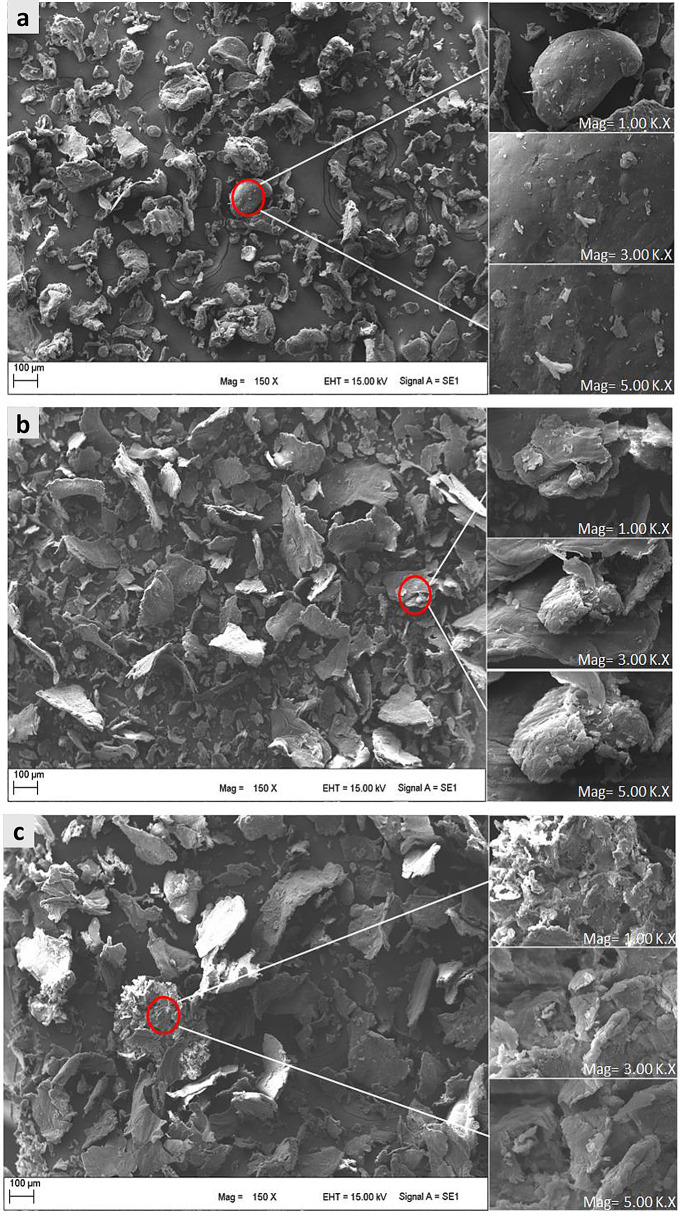
SEM images of CH (**a**), CHSB (**b**) and CCHSB (**c**) with a magnification of 150X, 3000X and 5000X

SEM micrographs revealed the structural characteristics and distribution patterns of chitosan and its derivatives. As illustrated in Fig. 1, the morphology of chitosan exhibited a generally smooth surface. In contrast, SEM images of CHSB demonstrated a rougher, and less ordered morphology compared to pure chitosan. This altered morphology is attributed to the formation of imine bonds (-C = N-) during the Schiff base reaction [35]. Notably, further modification of Schiff bases with cross-linking agents can induce changes in crystallinity, resulting in an amorphous appearance. The presence of small fibrils or sheet-like structures in CHSBs may also be linked to variations in thermal stability and solubility observed in CHSB and CCHSB.

### FTIR Analysis

**Fig. 2:**
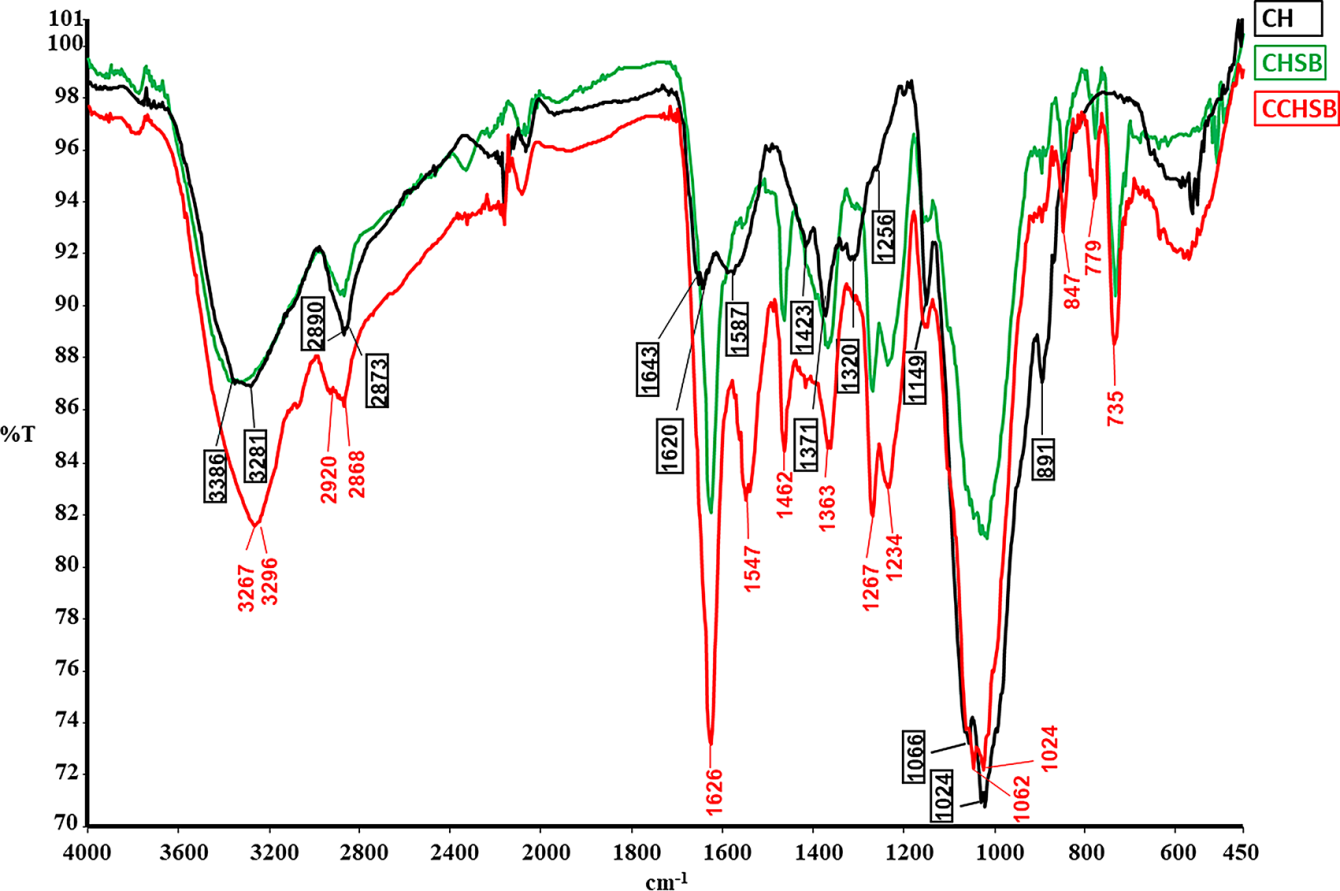
FTIR spectra of CH, CHSB and CCHSB

The FTIR spectra of CH, CHSB, and CCHSB are presented in Fig. 2. A comparative analysis of the spectra of CH and its derivatives multiple characteristic bands within the 4000–400 cm⁻¹ range. Broad absorption bands observed at 3281–3386 cm⁻¹ correspond to molecular hydrogen bonding as well as N-H and O-H stretching vibrations [36]. Bands in the 2920–2868 cm⁻¹ range were associated with ν(N-H) and ν(NH₂) stretching vibrations of primary amines [37-38]. The characteristic band at 1643 cm^− 1^ is assigned to the residual N-acetyl groups (C=O stretching of amide I) and 1320 cm^− 1^ (C-N stretching of amide III), respectively. A band at 1587 cm^− 1^ corresponds to the (N-H) bending of amide II. The CH_2_-bending and the CH3-symmetric deformations were confirmed by the presence of bands at 1423 and 1371 cm^− 1^, respectively [39]. Peaks observed at 1149 cm⁻¹ and 891 cm⁻¹ correspond to the β (1→4) glycosidic linkage and CH₂ bending due to the pyranose ring, respectively. Additionally, the band at 1062 cm⁻¹ was assigned to C-O stretching [40] and a small signal at 1256 cm⁻¹ indicated the bending vibrations of OH groups in chitosan [41]. In the FTIR spectrum of the Schiff base derivatives, the appearance of a peak corresponding to the imine group (-C = N-) in the range of 1234–1626 cm⁻¹ confirmed the formation of Schiff base compounds. Furthermore, a band at 735 cm⁻¹ was attributed to the bending vibrations of aromatic CH rings.

### ^1^H- NMR Analysis

**Fig. 3:**
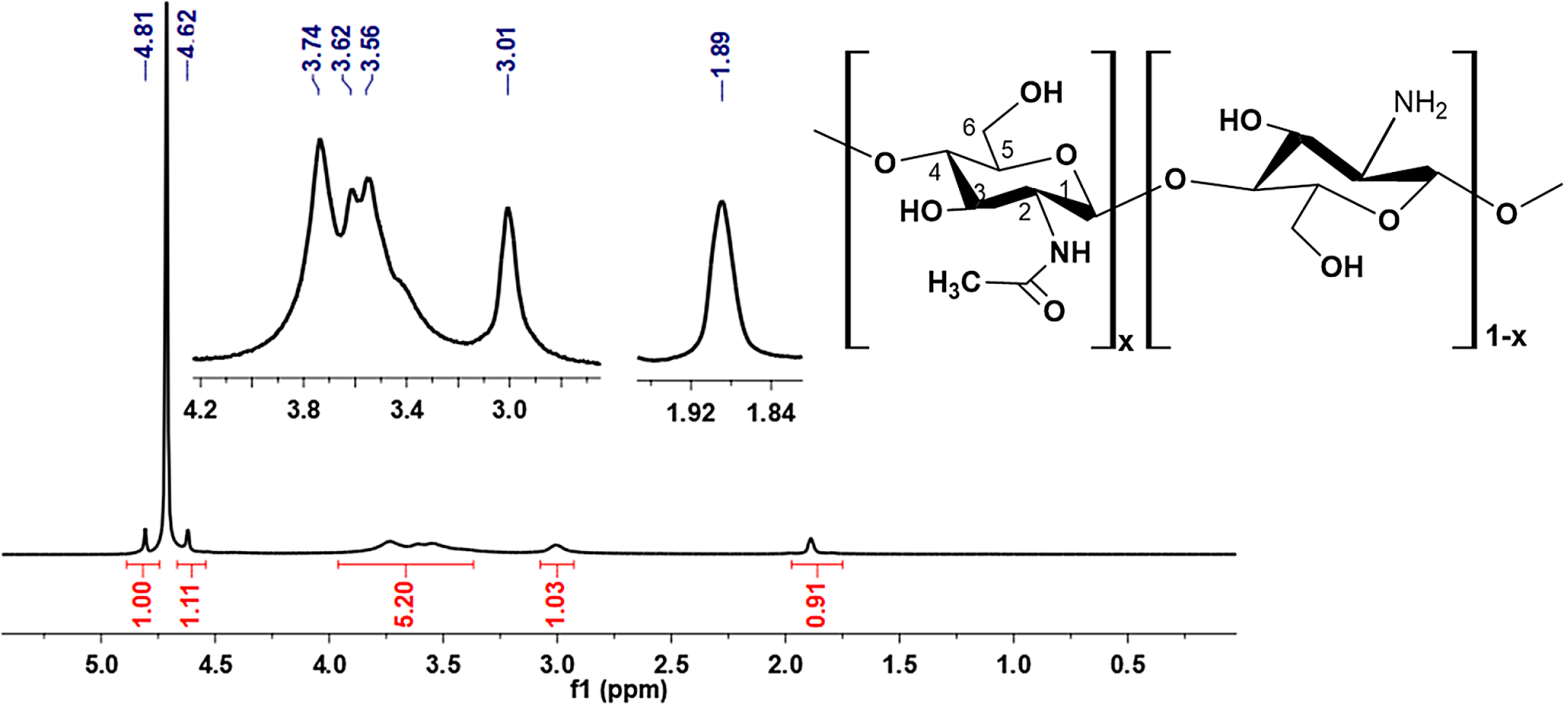
^1^H NMR spectra of CH

The ^1^H NMR spectrum of the CH is recorded in CD_3_COOD/D_2_O solution and presented in Fig. 3. The peak at 1.89 ppm corresponds to hydrogen atoms in the acetyl group, reflecting the presence of residual N-acetyl (-N(CO)CH₃) groups in the polymer due to the 75–85% degree of deacetylation. Complete removal of acetyl groups as a result of deacetylation of chitin is not possible due to the polymeric structure. This situation highlights the limitation of achieving complete deacetylation of chitin due to its polymeric structure. The signal at 3.01 ppm is assigned to the C2 hydrogen atom of the glucosamine ring, while signals between 3.56 and 3.74 ppm are attributed to the hydrogens at positions C4, C6, C3, and C5 of the chitosan backbone. The peak at 4.62 ppm is attributed to hydrogen in OH groups [35, 40–43]. The peak at 4.81 ppm is attributed to the hydrogen in the C1 group.

**Fig. 4:**
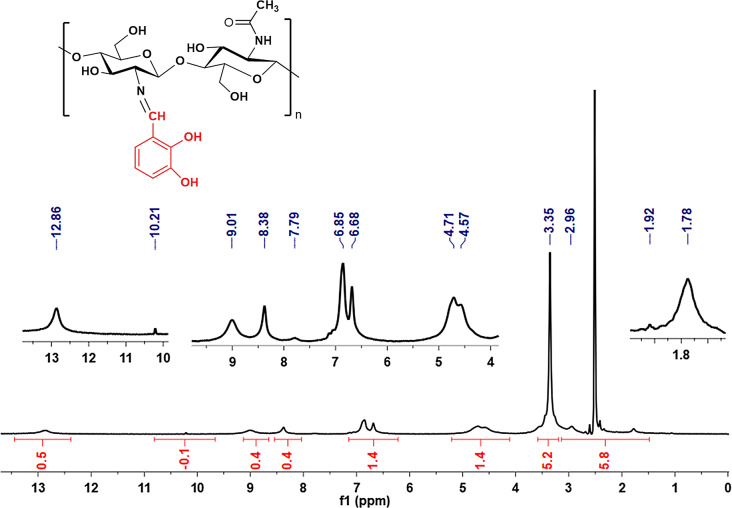
^1^H NMR spectra of CHSB

The ^1^H-NMR spectrum of the CHSB obtained from the reaction between chitosan and 2,3-dihydroxybenzaldehyde is recorded in CD_3_COOD/D_2_O solution and given in Fig. 4. The characteristic chemical shifts ​​of chitosan were observed between 1.78 and 4.71 ppm. The strong signal observed at 3.35 ppm is due to methylene (C-5-CH2) protons bound to the chitosan ring. The peaks between 6.68 and 7.79 ppm are attributed to the aromatic ring protons of the chitosan Schiff base. The signal seen at 8.38 ppm belongs to the azomethine group. The signals observed in the spectrum at 12.86 and 10.21 ppm originate from phenolic hydroxy groups.

**Fig. 5:**
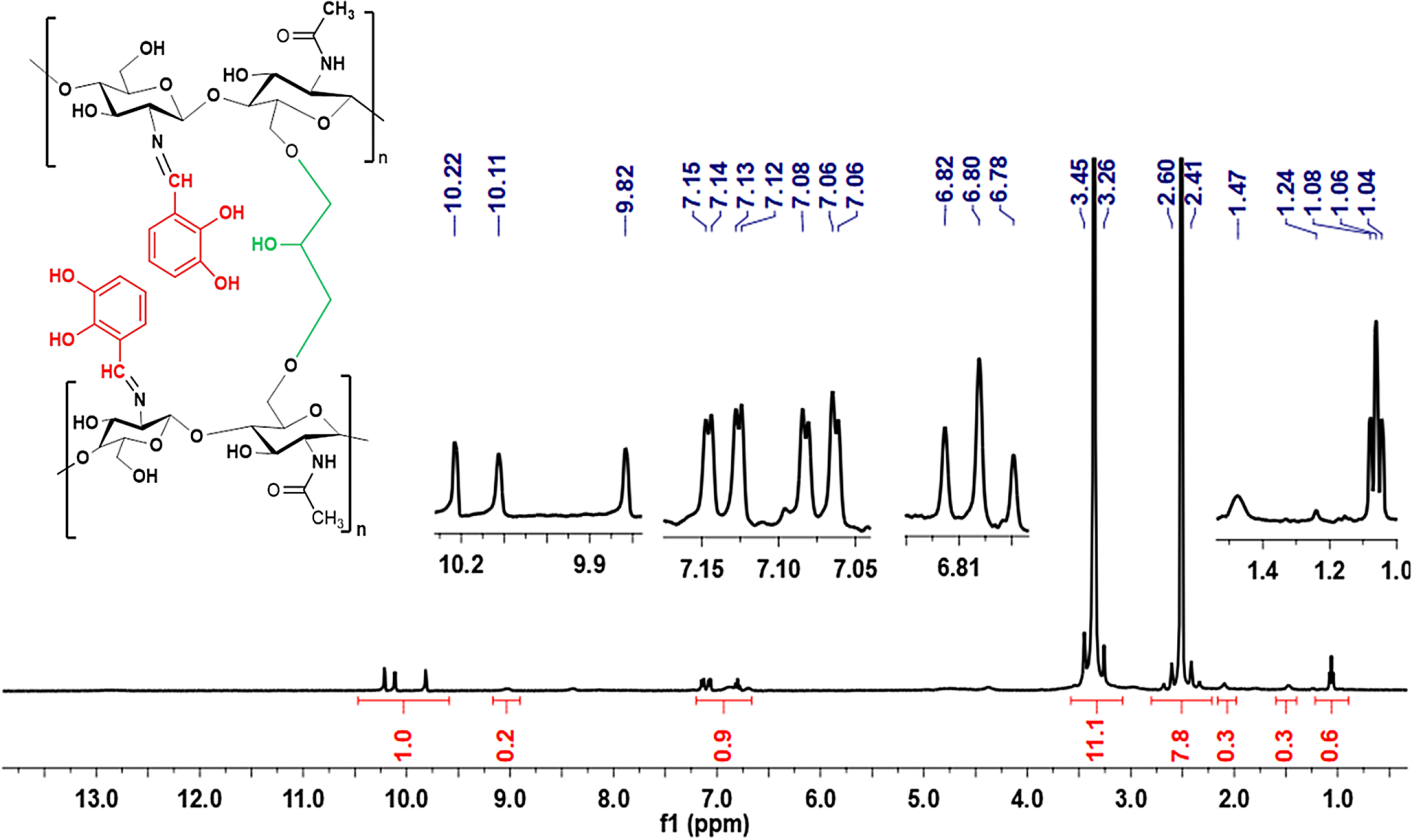
^1^H NMR spectra of HECH

The ^1^H-NMR spectrum of the CCHSB obtained from the reaction between chitosan, 2,3-dihydroxybenzaldehyde and epichlorohydrin i recorded in CD_3_COOD/D_2_O solution and given in Fig. 5. The chemical shifts observed in this spectrum are similar to those in the CHSB spectrum, with minor variations attributed to the cross-linking process. The signals corresponding to the epichlorohydrin methylene protons, involved in the cross-linking, are observed as triplets in the 1.24–1.04 ppm range.

### XRD Analysis

**Fig. 6:**
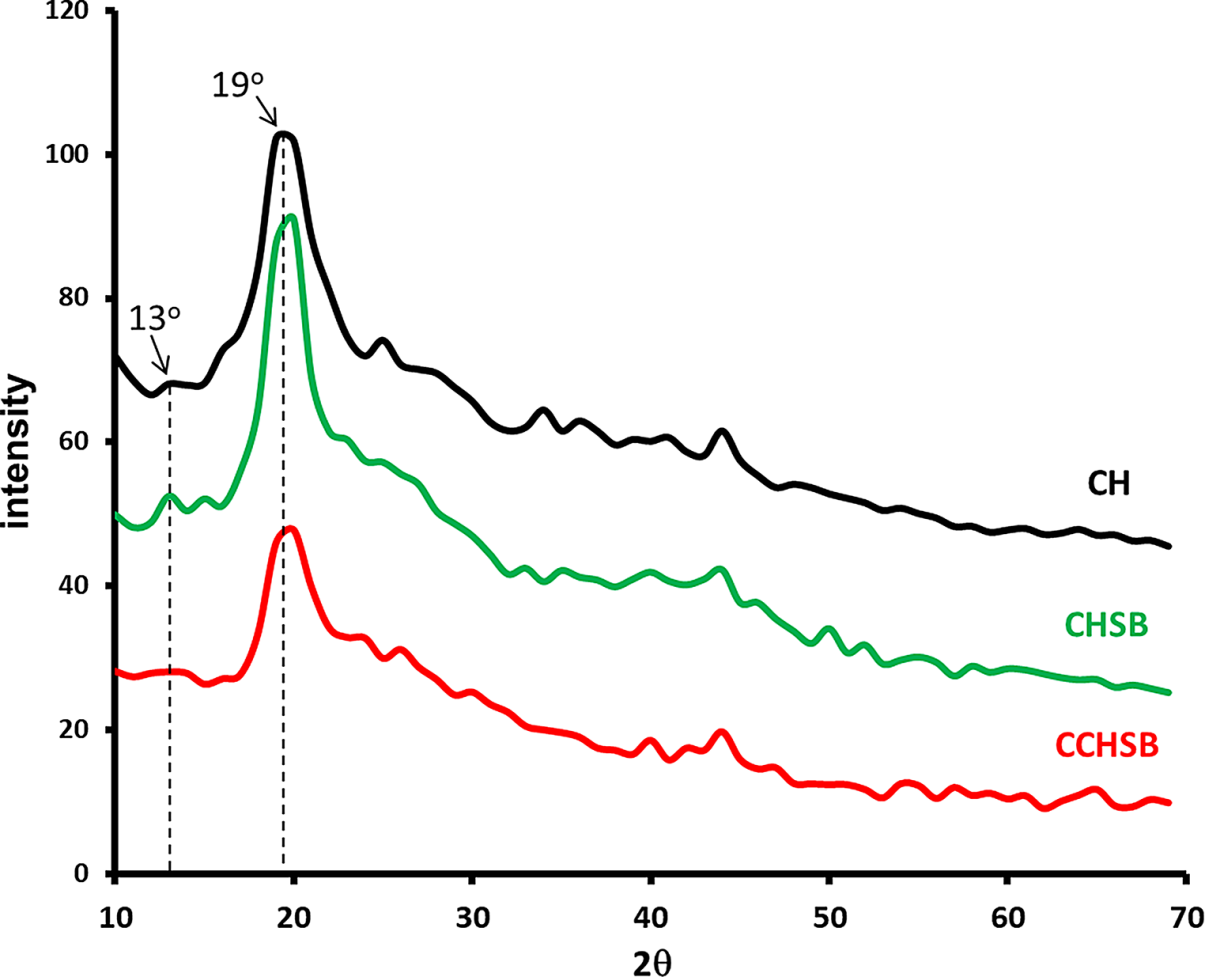
XRD pattern of CH, CHSB and CCHSB

The crystallinity of CH, CHSB, and CCHSB is shown in Fig. 6. Characteristic diffraction peaks for chitosan were observed at 2θ = 13° and 2θ = 19°, corresponding to crystalline regions in the polymer [37]. These results suggest that the polymer chain structure of chitosan is relatively ordered, with the hydroxyl and amino groups forming strong hydrogen bonds. The weak peak around 2θ = 14° (amine I,–N–CO–CH_3_) and the peak at 2θ = 20° (amine II,–NH_2_) belong to the main crystal structure [44]. The XRD diffractions of CHSB obtained from the reaction between chitosan and 2,3-dihydroxybenzaldehyde and CCHSB obtained from the reaction between chitosan, 2,3-dihydroxybenzaldehyde and epichlorohydrin are examined, it is seen that no significant changes are observed in the crystal and amorphous regions of chitosan. However, the intensity of the peak at 2θ = 19° decreased in CCHSB, which is indicative of disruption in the chitosan hydrogen bonding due to the chemical reaction.

### TGA Analysis

**Fig. 7:**
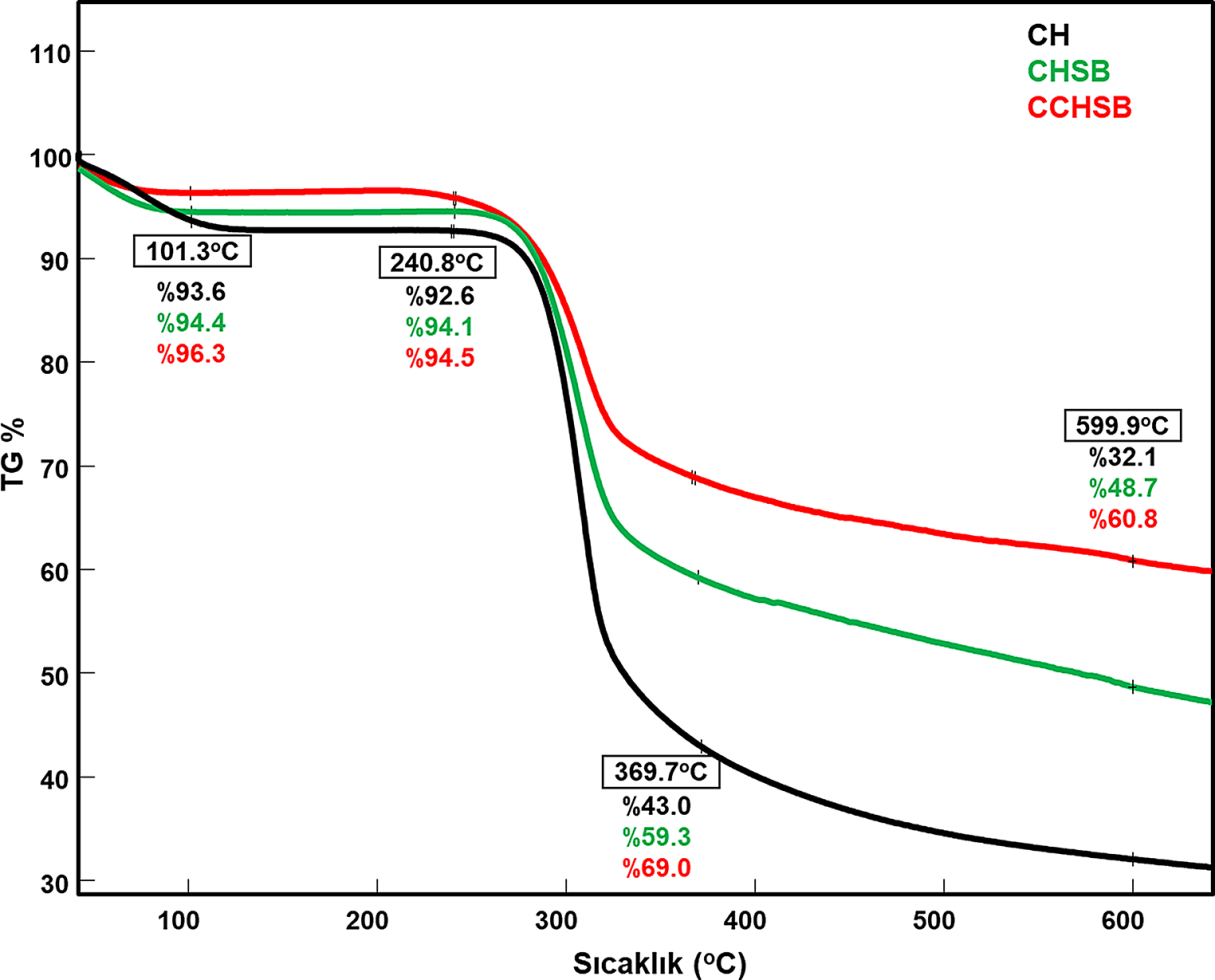
TG of CH, CHSB and CCHSB

Thermal properties of CH and CHSB and CCHSB were evaluated by thermogravimetric analysis. Thermogravimetric analysis is a process in which a material is degraded by heat, causing bonds within the molecule to break and plays an important role in determining the thermal stability of materials. The TGA curves of CH, CHSB and CCHSB are given in Fig. 7. Initial degradation was observed at approximately 101.3 °C, with a weight loss of 6.4% in CH, 5.6% in CHSB, and 3.7% in CCHSB, attributed to the loss of adsorbed or weakly hydrogen-bonded water from hydroxyl and amine groups. The main thermal degradation occurred between 240.8 °C and 369.7 °C due to the depolymerization of the polysaccharide and the resulting loss of volatile compounds. The total weight loss in this stage was 49.6% for CH, 34.8% for CHSB, and 25.0% for CCHSB. At 599.9 °C, the final weight loss was 67.9% for CH, 51.3% for CHSB, and 39.2% for CCHSB. When the thermal stabilities of CH, CHSB and CCHSB were compared, it was observed that CCHSB had the highest thermal stability, whereas CH exhibited the lowest.

### Antibacterial Evaluations

The Antibacterial activities of the CH, CHSB, and CCHSB were evaluated against both gram-negative bacteria *Escherichia coli* and *Salmonella Typhimurium*, and gram-negative bacteria *Staphylococcus aureus* and *Bacillus cereus*, and their antibacterial activities compared to that of the antibiotic’s amikacin and gentamicin. The results, summarized in Table 1, CH and Schiff base derivatives exhibited antibacterial activity against the *E. coli*, *S. typhimurium*, *S. aureus*, and *B. cereus* with inhibition zones ranging from 8 to 30 mm. The CHSB exhibited inhibition zones of 12 mm, 12 mm, 16 mm, and 16 mm against *E. coli*, *S. Typhimurium*, *S. aureus*, and *B. cereus*, respectively. The CCHSB showed inhibition zones of 8 mm, 12 mm, 12 mm, and 10 mm against *E. coli*, *S. Typhimurium*, *S. aureus*, and *B. cereus*, respectively. The results indicate that the CHSB has superior antibacterial activity compared to CH and CCHSB. This enhanced bioactivity can be attributed to the presence of Schiff base ligands, as previously reported in the literature [45–47]. These results highlight the potential of chitosan derivatives as effective antibacterial agents for various applications.

**Table 1:** The biological activity of the CH derivatives

Compounds	E. coli		S. Typhimurium		S. aureus		B. cereus	
	IZ (mm)	MIC (mg/mL)	IZ (mm)	MIC (mg/mL)	IZ (mm)	**MIC** (mg/mL)	IZ (mm)	MIC (mg/mL)
CH	8	1.25	12	1.25	12	1.25	22	1.25
CHSB	12	12.5	12	12.5	16	12.5	16	12.5
CCHSB	8	12.5	12	12.5	12	12.5	10	1.25
Amikasin	24		28		28		30	
Gentamisin	18		22		22		20	

IZ: inhibition zone

## Conclusion

This study successfully synthesized of chitosan derivatives: chitosan Schiff base and crosslinked chitosan Schiff bases. The physical appearances of the compounds were noted by visual observation. The materials obtained were brown and deep yellow powdered solids as presented in Scheme 1, except for chitosan. The synthesized compounds were characterized using various analysis techniques. This comprehensive characterization is crucial to understand the properties and potential applications of these compounds. The chitosan derivatives demonstrated significant antibacterial properties against *E. coli, S. typhimurium, S. aureus, and B. cereus*. The potential of chitosan derivatives as effective alternatives or adjuncts to conventional antibiotics such as amikacin and gentamicin can be significant to create new formulations that antibiotic resistance. Chitosan has unique properties such as being non-toxic, biocompatible, and able to interact positively with biological tissues. This makes it safe for medical use. In addition, chitosan derivatives, with their antibacterial activity, have the potential to improve food preservation in the food industry. They may serve as natural preservatives, helping to extend the shelf life of perishable products by inhibiting microbial growth and ensuring food safety and quality.

## Data Availability

No datasets were generated or analysed during the current study.
